# Dynamic profiling of immune microenvironment during pancreatic cancer development suggests early intervention and combination strategy of immunotherapy

**DOI:** 10.1016/j.ebiom.2022.103958

**Published:** 2022-03-19

**Authors:** Jiaqi Yang, Qi Zhang, Junli Wang, Yu Lou, Zhengtao Hong, Shumei Wei, Ke Sun, Jianing Wang, Yiwen Chen, Jianpeng Sheng, Wei Su, Xueli Bai, Tingbo Liang

**Affiliations:** a Department of Hepatobiliary and Pancreatic Surgery, The First Affiliated Hospital, Zhejiang University School of Medicine, No. 79 Qingchun Road, Hangzhou 310003, China; b Key Laboratory of Pancreatic Disease of Zhejiang Province, Hangzhou, China; c Innovation Center for the Study of Pancreatic Diseases of Zhejiang Province, Hangzhou, China; d Zhejiang Clinical Research Center of Hepatobiliary and Pancreatic Diseases, Hangzhou, China; e Cancer Center, Zhejiang University, Hangzhou, Zhejiang, 310058 China; f Department of Pathology, The Second Affiliated Hospital, Zhejiang University School of Medicine, Hangzhou, China; g Department of Pathology, The First Affiliated Hospital, Zhejiang University School of Medicine, Hangzhou, China

**Keywords:** Pancreatic ductal adenocarcinoma, Tumor microenvironment, Mass cytometry, Tumor-infiltrating immune cells, Immunotherapy

## Abstract

**Background:**

Pancreatic ductal adenocarcinoma (PDAC) has little response to immune checkpoint inhibitors. An in-depth understanding of the immune microenvironment from a comprehensive and dynamic perspective is critical to generate effective therapeutic strategies for PDAC.

**Methods:**

Using mass cytometry and immunohistochemistry, we explored the dynamic changes of tumor-infiltrating immune cells during the development of PDAC in a genetically engineered mouse model (Kras^G12D/+^; Trp53^R172H/+^; Pdx1-cre) and human specimens. PD-L1^−/−^ mice were crossed with Kras^G12D/+^; TgfβR2^flox/flox^; Ptf1a-cre mice to achieve early depletion of PD-L1 in pancreatic cancer. Combination therapy of Arginase-1 (Arg-1) inhibitor and anti-PD-1 mAb was validated in syngeneic mouse models.

**Findings:**

Two different stages of immunosuppression with unique features were observed in both mouse model and human specimens. Early stage of immunosuppression featured highly abundant Tregs during acinar-to-ductal metaplasia, despite of a prominent and continuous presence of effector lymphocytes. The differentiation/activation branch of Ly-6C^+^ monocytes changed from a BST2^+^/MHC-II^+^ phenotype to an Arg-1^+^ phenotype over time during PDAC development. The late stage of immunosuppression thus featured the presence of a large number of myeloid suppressive cells together with a significant reduction of effector lymphocytes. Removal of PD-L1 from the beginning efficiently triggered anti-tumor immunity and significantly prolonged survival in PDAC-developing mice. Targeting Arg1^+^ macrophages with an Arg-1 inhibitor synergized with anti-PD-1 immunotherapy and led to PDAC-specific immune memory.

**Interpretation:**

By demonstrating the coevolution of histopathology and immunology in PDAC, this study highlights the necessity and value of early intervention and combinational approach in leveraging immunotherapy to treat pancreatic cancer.

**Funding:**

A full list of funding bodies that contributed to this study can be found in the Acknowledgements section.

**Research in context**

**Evidence before this study**

Most clinical trials involving patients with advanced pancreatic cancer have failed, which is often thought to be due to pancreatic cancer's immune desert microenvironment. Whether pancreatic cancer remains consistently “nonimmunogenic” is unclear. Resently, neoadjuvant immunotherapy for resectable pancreatic cancer has been reported to significantly induce T cell infiltration, activation and proliferation. Therefore, it is necessary to explore the dynamic changes of immune microenvironment in pancreatic cancer to determine whether there is a window period suitable for immunotherapy.

**Added value of this study**

This study demonstrated the dynamic evolution of the immune microenvironment during the development and progression of pancreatic cancer. The immunosuppression of pancreatic cancer gradually aggravates and eventually forms the myeloid cell-dominated immune microenvironment. Early pancreatic cancer still has a large number of active lymphocytes, which may be the window period for immunotherapy.

**Implications of all the available evidence**

This is the first study to delineate the dynamic immune landscape of pancreatic cancer, covering the entire histopathological progression. The changes of immune microenvironment in pancreatic cancer and the immune characteristics at different stages can provide a reference for the development of immunotherapy strategies.

Alt-text: Unlabelled box

## Introduction

Pancreatic ductal adenocarcinoma (PDAC) is the most lethal form of cancer and the prognosis of PDAC patients has not substantially improved in recent decades.^1^ Two chemotherapeutic regimens (i.e., FOLFIRINOX and gemcitabine plus nab-paclitaxel) have shown some survival benefit^2^^,^^3^; however, PDAC remains largely incurable. With immunotherapy advances in other solid tumors, several attempts have been made to treat PDAC with immune checkpoint inhibitors, tumor vaccination, and chimeric antigen T cells; however, no definite improvement in survival has been observed.^4^, ^5^, ^6^, ^7^ The local immunity of PDAC largely determines the efficacy of immunotherapy,^8^ whereas the understanding of the immune microenvironment is far from sufficient.^9^ Therefore, elucidation of PDAC immunology is critical for the development of more effective immunotherapeutic strategies.

Both the local and systemic immune system are capable of clearing cancerous cells in immune competent individuals, and successful immunotherapy is dependent on the infiltration of sufficient effector cells.^10^ However, PDAC often presents at an advanced stage and is frequently characterized by an abundance of immune regulatory cells.^11^ A suppressive local immunotype can contribute to tumor progression and impede the success of immunotherapy.^12^ It is suggested that early intervention with immunotherapy appears to be more effective for activating the anti-tumor immune response.^13^ When the local immunity is gradually compromised and an acquired immune suppressive microenvironment is established instead, the question of whether there is a therapeutic window for immunotherapy during PDAC evolvement must be addressed. To resolve this issue, a comprehensive immune landscape with dynamic characteristics is warranted.

It is impossible to trace the dynamic changes in the tumor immune microenvironment from a normal pancreas to metastatic PDAC in human patients. Thus, we studied a spontaneous PDAC mouse model that harbors similar somatic alterations with human PDAC using mass cytometry (CyTOF), and verified the findings in human patients with different stages of the disease using immunohistochemistry (IHC). Our findings revealed that the immune landscape varied in mice exhibiting different histopathological stages, and we demonstrated that removal of programmed cell death-ligand 1 (PD-L1) signaling from the beginning effectively activated the anti-tumor immune response and prolonged survival time in PDAC-developing mice. Targeting myeloid-derived immunosuppression in PDAC with an arginase-1 (Arg-1) inhibitor demonstrated a profound synergy with immune checkpoint inhibitors. Additionally, we confirmed that there was a high consistency of immune variability in PDAC between the mouse model and human patients. Thus, the early intervention and combination strategy may also bring potential therapeutic enhancement to human patients. Overall, we uncovered the dynamic immune landscape that occurs throughout the development of PDAC and provides a fundamental reference of the immune characteristics at different stages of PDAC to develop promising immunotherapeutic strategies.

## Methods

### Mice

**Figure 1:**
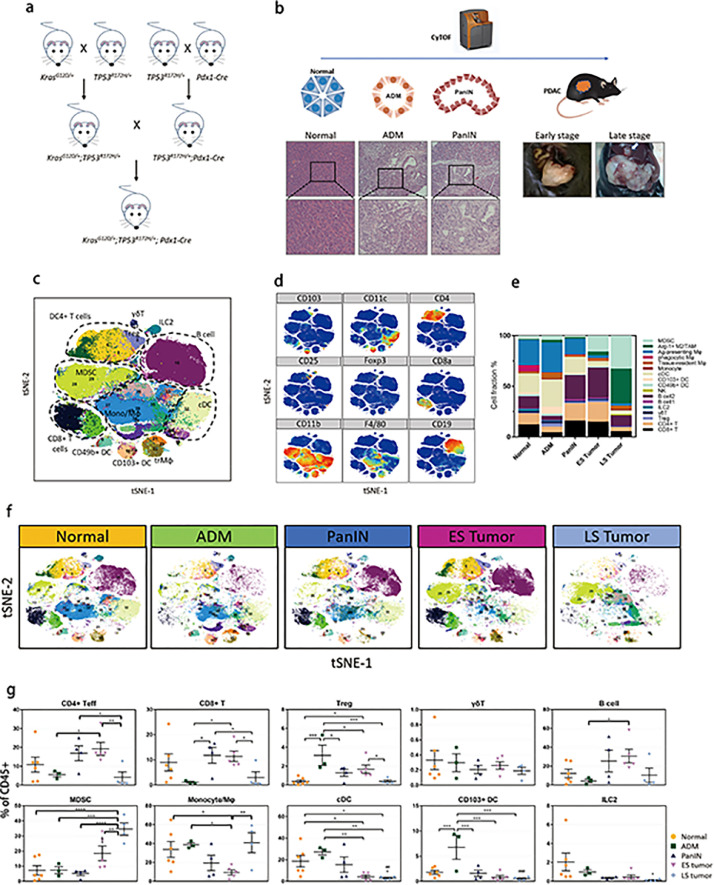
Intratumoral immune cell profiling by CyTOF. (a) Breeding stragety of KPC mice. (b) Five stages of PDAC development. Precancerous stages were identified by histopathology and tumor stages were divided by whether metastasis exists or not. Fresh samples were stained and detected by CyTOF. (c, d) tSNE analysis of the total immune cell populations according to some specific markers. (e, f) The immune contexture in the five developmental stages. (g) Percentage of each immune cell populations (mean percent ± SD of total immune cells), * *p* < 0.05, ** *p* < 0.01, *** *p* < 0.001, one-way ANOVA multiple comparisons. ES Tumor, early-stage tumor; LS Tumor, late-stage tumor.

The *LSL-Kras^G12D^, LSL-Trp53^R172H^*, and *Pdx1-Cre* genetically engineered mice (purchased from the Jackson Laboratory, Stock Nos. 008179, 008652, and 014647) were crossed as described in Figure 1a to generate KPC mice. To obtain normal and precancerous samples, KPC mice were sacrificed at 1–4 months after birth, respectively. PDAC samples were harvested from KPC mice that exhibited palpable lesions. Metastasis in the liver, lung, peritoneum, as well as other organs was investigated. Each sample was divided into two parts: (1) one part was fixed using formalin and embedded with paraffin; and (2) the other part was kept fresh for CyTOF analysis. KTC mice were obtained from professor Hideaki Ijichi and Harold L Moses as a gift,^14^ KTC mice were crossed to PD-L1^−/−^ mice (purchased from the Jackson Laboratory, Stock No. 012675).

### Cell lines

KPC tumor-derived cell line (KPC cell) was separated from spontaneous KPC tumor and was identified as a genotype of Kras^G12D/+^; Trp53^R172H/-^ by standard PCR. Mouse melanoma cell line B16F10 and PancO2 were purchased from the American type culture collection (ATCC) in 2017 and were validated by Short Tandem Repeat authentication. The cells were cultured in RPMI 1640 medium (KPC and PancO2) or DMEM medium (B16F10) with 10% fetal bovine serum and 1% penicillin-streptomycin.

### Syngeneic model

6-week old male C57BL/6 mice were purchased from Model Organisms (Shanghai, China). 5 × 10^5^ KPC or PancO2 cells in 100 μL PBS were subcutaneously injected into the right flank of C57BL/6 mice. About 7 days post tumor injection, when the tumors grew to an average size of 35 mm^3^, the mice were randomly divided into 4 groups. Arg-1 inhibitor was administered daily by oral gavage at 30 mg/kg (Cat# HY-15775, MCE, NJ, USA) and anti-PD-1 monoclonal antibody (mAb, 200 μg, clone RMP1-14, BioXcell, West Lebanon, USA) was injected intraperitoneally twice per week. The control mice were treated with PBS and IgG isotype. Mice were euthanized when tumor necrotized or volumes reached 800 mm^3^.

### Tumor rechallenge

Mice which had rejected KPC tumor (the mice were free of tumor) after treatment with anti-PD-1 antibodies and Arg-1 inhibitor were rechallenged with KPC and B16F10 cells (10^6^ cells in 100 μL PBS) subcutaneously in the left and right flank, respectively. Naïve mice were engrafted with KPC and B16F10 cells in the same manner as a control. Tumor volumes were measured once every two days with a digital caliper.

### Patient samples

Formalin-fixed paraffin embedded (FFPE) samples were obtained from the Department of Hepatobiliary and Pancreatic Surgery, the First Affiliated Hospital of Zhejiang University School of Medicine (FAHZU). The pancreas samples were obtained from organ donors for liver transplantation, and the PDAC samples were obtained from patients who underwent surgical resection or an aspiration biopsy. The study was approved by the ethics committee of FAHZU, and all patients formally consented. The clinical information of the pancreas donors and PDAC patients was summarized in Tables S5 and 6. The disease stage was diagnosed according to the National Comprehensive Cancer Network Clinical Practice Guidelines for Pancreatic Adenocarcinoma (Version 1.2020).

### Pathological review

Judgement of the pathological stage of mouse pancreatic tissues was independently performed by two senior pathologists who specialized in PDAC. For the samples with inconsistent results, full discussion with another author was performed until the three experts reach a consensus. Briefly, a normal pancreas was defined when only a regular acinar structure was observed; acinar-to-ductal metaplasia (ADM) was confirmed with the pathological features of the remaining lobular contour of the pancreatic acinus and replacement of acinar structure by ductal structure without atypia; and pancreatic intraepithelial neoplasia (PanIN) was reported for a papillary sample, with a loss of polarity, pseudostratified, or stratified nuclei, cytological atypia, and mitosis. In the case of PDAC, local disease was defined as early stage, whereas late stage was diagnosed when the tumor involvement of the other organs was observed and histologically confirmed. In case that more than one morphology was observed, we would classify it as the more malignant stage (e.g., if ADM and PanIN were both identified, we classify the sample as PanIN stage). The timeline of PDAC development was established according to the histological results.

### CyTOF samples and antibodies

Murine samples were dissociated into small pieces using sterile scissors followed by enzymatical digestion with RPMI 1640 containing 2 mg/mL collagenase, 250 μg/mL hyaluronidase, and 20 μg/mL DNase I for 1 h at 37 °C. An average of 2 × 10^6^ cells per sample was obtained for subsequent staining and analysis. Either pre-conjugated or purified antibodies were purchased from Fluidigm (South San Francisco, CA, USA). Purified antibodies were further conjugated using MaxPar X8 Polymer Kits (Fluidigm) according to the manufacturer's instructions. The mass cytometry antibodies for the 42 markers used in this study are listed in Tables S1 and S2.

### CyTOF staining and data acquisition

A total of 1 × 10^6^ cells in each sample were blocked (BioLegend, San Diego, CA, USA) and stained for cell surface markers in staining buffer (PBS containing 0.5% BSA and 0.02 % NaN_3_) for 30 min at 4 ℃. For intracellular and intranuclear staining, the cells were fixed with fixation/permeabilization buffer for 15 min. The cells were then washed and stained in the transcriptional factor staining buffer according to the manufacturer's instructions (BD Biosciences, San Jose, CA, USA). The samples were fixed again in 1.6% paraformaldehyde and stained with DNA intercalator iridium overnight at 4 ℃. Cells were then acquired using a Helios instrument after adding normalization beads. FCS files were concatenated and normalized with beads using Helios software (Fluidigm).

### CyTOF data analysis

Mass cytometry data were de-barcoded using a doublet filtering scheme with mass-tagged barcodes. Live, singlet, and valid immune cells were manually identified. The X-shift (Phenograph) algorithm was run to obtain accurate immune subset information for all the samples. Markers used to identify the cell populations were summarized and listed in Table S3. GraphPad Prism 7 software (San Diego, CA, USA) was used to perform the comparison and correlation analyses. A pseudotime analysis was performed using the Monocle3 R package. Some lineage-relative markers were used to create the trajectory graph.

### Immunohistochemistry

Slides were cut from FFPE samples at a thickness of 4 μm. After blocking endogenous peroxidase and antigen retrieval, the slides were then stained using anti-CD206 (1:1000, Cat# ab64693, Abcam, Cambridge, MA, USA), anti-FoxP3 (1:100, Cat# 12653S, CST for mouse; 1:100, Cat# ab20034, Abcam for human), anti-CD3 (1:250, Cat# ab11089, Abcam), anti-CD11b (1:800, ab133357, Abcam), anti-CD11c (1:150, Cat# ab52632, Abcam), anti-CD19 (1:250, Cat# ab134114, Abcam), and anti-CD163 (1:500, Cat# ab189915, Abcam). Corresponding secondary antibodies (1:500, Cat# KIT-5003, KIT-5006, MXB Biotechnologies, Fuzhou, China) were used to incubate the slides followed by chromogenic DAB staining (Cat# GK347011, Gene Tech, Shanghai, China). All slides were enclosed with neutral resin following nuclear staining and observed with an Axio Imager M2 microscope (Zeiss, Oberkochen, Baden-Württemberg, Germany). The images were acquired with Zeiss ZEN software and the immune cells were quantified by counting three random high-power fields (400×) with relatively dense staining for each slice.

### Ethics

All the animal experiments were performed in compliance with the Laboratory animal-Guideline for ethical review of animal welfare (GB/T 35892-2018) and were approved by the Animal Experimental Ethical Inspection of FAHZU. The use of patient samples was approved by the ethics committee of FAHZU, and all patients formally consented.

### Statistics

A Student's *t*-test was used for comparisons between two groups. A one-way ANOVA was used to compare the differences among the five groups. A linear regression was used to fit the lines and quantify the relation degree of two variables. A Pearson's correlation coefficient was used to evaluate the correlation matrix. A Kaplan-Meier analysis was used to compute the patient survival curves and Log-rank tests were used to perform the comparison.

### Role of the funding source

Funders provide financial support for this study, and do not participate in study design, data collection, data analyses, interpretation, or writing of report.

## Results

### Comprehensive profiling of the immune microenvironment during PDAC initiation and progression

*KRAS* and *TP53* are the most frequent somatic mutations in Chinese and Western cohorts of human pancreatic cancer.^15^^,^^16^ To recapitulate human PDAC, we generated KPC mice by crossing three strains of genetically engineered mice (*LSL-Kras^G12D/+^;LSL-Trp53^R172H/+^;Pdx1-Cre*) (Figure 1a). The KPC mice developed spontaneous PDAC through acinar-to-ductal metaplasia (ADM) and pancreatic intraepithelial neoplasia (PanIN) at a median time of three months, and had a median survival time of six months.^17^, ^18^, ^19^ A total of 22 KPC mice were sacrificed at different time points to harvest their pancreatic tissues (Table S4). These samples were classified into five groups according to the pathological grade and disease stage upon gross and microscopic investigation (Figure 1b). CyTOF data from all the samples were pooled together for cell clustering, and 40 subtypes of immune cells were identified in the tumor microenvironment (Figure 1c and d). We observed significant variability in the immune microenvironment at different stages according to the constitution and abundance of the cell subtypes (Figure 1e and f). The ADM and late-stage tumor groups demonstrated an obvious “immunologically devoid” tumor microenvironment, which was characterized by minimal infiltration of lymphoid cells (Figure 1f). The proportion of both T cells and B cells was extremely low in both groups, which suggested compromised adaptive immunity. However, T regulatory cells (Tregs) and myeloid-derived suppressive cells (MDSCs) exhibited different trends. The proportion of Tregs increased transiently in the ADM stage and continuously decreased in subsequent stages. In contrast, MDSCs were dominant in the tumor stages, especially in late-stage tumors (Figure 1g). These results suggest that Tregs and MDSCs might contribute to the immunosuppression of the ADM pancreas and late-stage tumors, respectively, and played a key role in PDAC initiation and progression.

### Dynamic changes in lymphocytes during PDAC development

**Figure 2:**
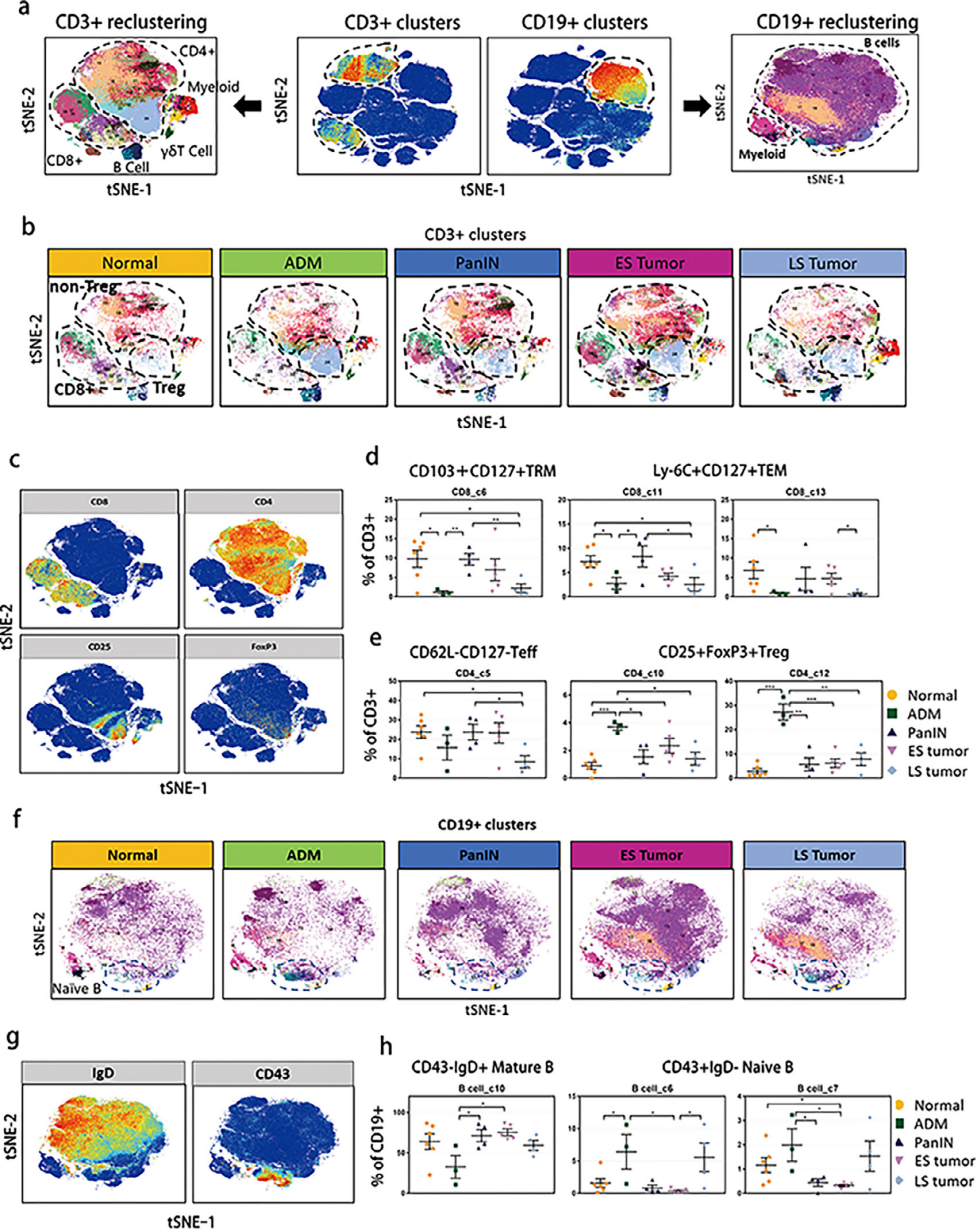
Characterization of intratumoral lymphocytes. (a) tSNE plots of CD3^+^ and CD19^+^ lymphoid cell populations. (b) tSNE plots displaying dynamic change of CD8^+^ T cells, CD4^+^ non-Tregs and Tregs in the five developmental stages. (c) tSNE plots identifying T cells with markers of CD8, CD4, CD25 and FoxP3. (d, e) Percentage of CD8^+^ and CD4^+^ T cells in clusters with significant change during PDAC development (mean percent ± SD of total T cells), * *p* < 0.05, ** *p* < 0.01, *** *p* < 0.001, one-way ANOVA multiple comparisons. (f) tSNE plots displaying dynamic change of B cells in the five developmental stages. (g) tSNE plots identifying B cells with markers of IgD and CD43. (h) Percentage of CD43^+^IgD^−^-immature and CD43^−^IgD^+^-mature B cell clusters during PDAC development (mean percent ± SD of total CD19+ B cells), * *p* < 0.05, ** *p* < 0.01, *** *p* < 0.001, one-way ANOVA, multiple comparisons.

For a detailed analysis, we reclustered T cells, B cells (Figure 2a). All of the CD3^+^ cells were manually gated and reclustered to detect 13 subclusters of CD8^+^ T cells and 12 subclusters of CD4^+^ T cells (Figure S1a and b). Three of the largest CD8^+^ subclusters (CD8_c6, CD8_c11, and CD8_c13) were substantially decreased in both the ADM pancreas and late-stage tumors (Figure 2b–d). The CD8_c6 subcluster expressed high levels of CD103 and CD127, exhibiting a resident memory T cell (T_RM_) phenotype. The CD8_c11 and CD8_c13 subclusters expressed high levels of Ly6C and CD127 (Figure S1a), which were phenotypically correspond to effector memory T cells (T_EM_).^20^^,^^21^ The percentage of other CD8^+^ subclusters (CD8_c1-5, CD8_7-10, and CD8_c12) were not significantly different among the five stages (Figure S1c).

Among the 12 subclusters of CD4^+^ T cells, the CD4_c10 and CD4_c12 subclusters increased in the ADM stage (Figures 2e and S1d). The two subclusters expressed relatively high levels of CD25, FoxP3, CD38, and ICOS, which indicated an active Treg phenotype. The dynamic alteration of Tregs analyzed from the reclustering of CD3^+^ cells was greatly consistent with the results without reclustering (Figure 2b and c), further highlighting its unique role in the ADM stage. In contrast, the abundance of the CD4_c5 subcluster, characterized by negative expression of CD62L and CD127, was decreased in both the ADM pancreas and late-stage tumors. The changing pattern of the CD4_c5 subcluster occurred in parallel with that of the CD8^+^ T_RM_ and T_EM_ subclusters, suggesting an anti-tumor role of these CD4^+^ cells (Figure 2c–e). Due to lack of some effector molecules (e.g. IFN-γ, IL-4, IL-10 and IL-17), we could not distinguish the subpopulations of Th1, Th2 and Th17 in the other clusters.

We unexpectedly detected a large number of B cells in the KPC mouse samples. Unfortunately, the tumor-infiltrating B cells were not well divided after the reclustering of CD19^+^ cells using our CyTOF antibody panel due to the limited number of channels (Figures 2a and S1h). Only two main subpopulations were identified based on CD43 and IgD expression (Figure 2g). A trajectory analysis showed that CD43^+^ subsets primarily consisted of a group of immature cells, whereas IgD^+^ subsets were more similar to mature cells (Figure S1–g). Intriguingly, we found that the proportion of CD43^+^IgD^−^ B cells increased in both the immunosuppressive stages in contrast to that of the CD43^−^IgD^+^ B cells (Figure 2f and h). The largest subcluster of CD43^−^IgD^+^ B cells (B cell_c10), which showed a low level in samples with ADM and late-stage tumor stages (Figure 2f and h), was potentially pro-inflammatory rather than immunosuppressive.

Taken together, these data showed that CD3^+^ T cells as well as CD19^+^IgD^+^ B cells exhibiting effector phenotypes were remarkably decreased in the ADM and metastatic tumor stages, playing an immunoactive role, while immunosuppressive Tregs exclusively accumulated in the ADM pancreas.

### Temporal changes in myeloid cells during PDAC development

**Figure 3:**
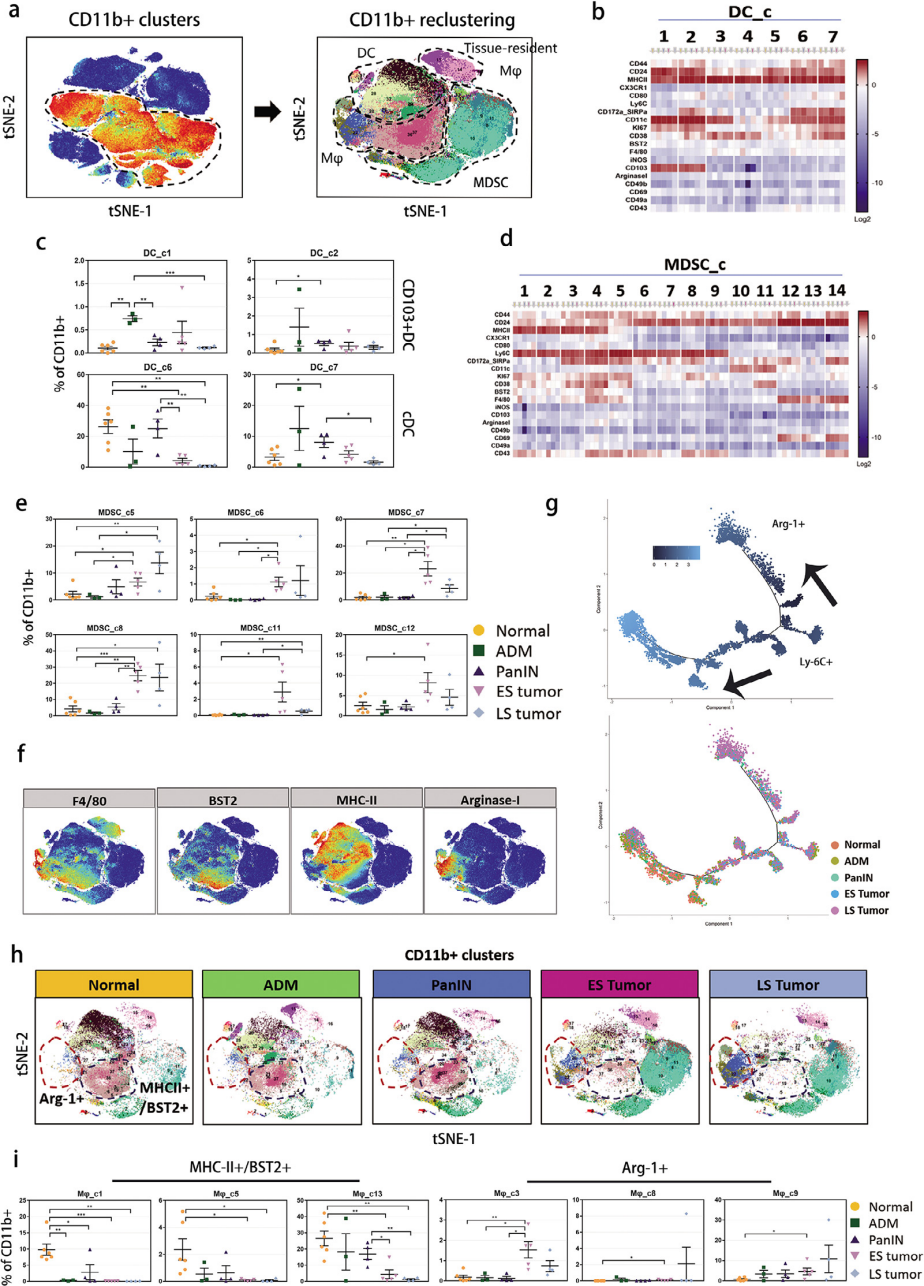
Characterization of intratumoral myeloid cells. (a) tSNE plots of CD11b^+^ myeloid cell populations. (b, d) Heatmap of DC and MDSC clusters with normalized expression of selected markers. (c, e) Percentage of DCs and MDSCs in each cluster (mean percent ± SD of total myeloid cells), * *p* < 0.05, ** *p* < 0.01, *** *p* < 0.001, one-way ANOVA multiple comparisons. (f) tSNE plots identifying distinguishable macrophage subtypes by markers of F4/80, BST2, MHC-II and Arg-1. (g) Trajectory analysis of the time course of monocytes/macrophages differentiation/activation. (h) tSNE plots of Arg-1^+^ and MHC-II^+^/BST2^+^ macrophages in the five developmental stages. (i) Percentage of MHC-II^+^/BST2^+^ macrophage AND Arg-1^+^ macrophage clusters (*p < 0.05, ** *p* < 0.01, *** *p* < 0.001, one-way ANOVA multiple comparisons.

The reclustering of CD11b^+^ cells revealed four main cell types: (1) dendritic cells (DCs); (2) macrophages; (3) CD69^+^ tissue-resident macrophages; and (4) monocytic MDSCs (Figure 3a). We identified seven subclusters of DCs, among which the DC_c1 and DC_c2 subclusters were CD103^+^, which could be distinguished from the DC_c6 and DC_c7 subclusters (CD103^−^CD172a^+^) (Figure 3b). All of the DCs expressed high levels of major histocompatibility complex II (MHC-II), indicating an antigen-presenting function. The DC_c1 and DC_c2 subclusters had particularly accumulated in the ADM pancreas (Figure 3c). The DC_c6 and DC_c7 subclusters comprised the dominant proportion and displayed a progressive decrease along with the evolving tumor. The decrease in DCs with the tumor stages suggested that there was an insufficient antigen-presenting function to induce anti-tumor immunity.

MDSCs comprise a mixture of different types of immature myeloid cells that lead to immunosuppression.^22^ We identified five subclusters of monocytic MDSCs (MDSC_c5-9), which were F4/80^−^Ly6C^+^MHC-II^−^ (Figure 3d). We also observed another two subpopulations of MDSCs, which were CD11c^+^MHC-II^−^ (MDSC_c10 and c11) and F4/80^+^CD69^+^MHC II^−^ (MDSC_c12-14), respectively. The other subclusters (MDSC_c1-4) were likely to be monocytes that expressed MHC-II, lymphocyte antigen 6 complex, locus C1 (Ly6C) and moderate levels of F4/80. Nearly all of the MDSC subsets rapidly accumulated after tumor formation (Figures 1g and 3e), which suggested that MDSCs may have dominated the immunosuppressive tumor microenvironment in PDAC.

### Macrophages undergo a functional shift in line with PDAC development

Macrophages comprised a large portion of infiltrating myeloid cells in mouse PDAC (Figure 1a and c). The phenotype and function of macrophages is dependent on the stimuli received from the tumor microenvironment.^23^ Our data showed that during PDAC development, the macrophages were phenotypically divided into subgroups of BST2^+^/MHC-II^+^ (Mφ_c1, c5-7, and c10-14) and Arg-1^+^ (Mφ_c2-4, c8, and c9) (Figures 3f and S2c). BST2^+^ macrophages have been reported to play an important role in restricting viral infection,^24^^,^^25^ which indicates an active innate immune status. MHC II expression represents the antigen-presenting capacity of macrophages to trigger a CD4^+^ T cell response.^26^ To study the functional repolarization of macrophages during PDAC carcinogenesis and progression, we conducted a pseudotime analysis for all monocytes and macrophages. The trajectory graph suggested that the Ly6C^+^ monocytes were determined to become either BST2^+^/MHC-II^+^ or Arg-1^+^ macrophages during cell development (Figure 3g). In addition, as the pancreas changed from normal tissue to late-stage PDAC, the Ly6C^+^ monocytes preferentially developed toward the Arg-1^+^ orientation (Figure 3g). Representative subclusters (Mφ_c1, c5, and c13) of these potential anti-tumor BST2^+^/MHC-II^+^ macrophages decreased and even disappeared when the tumor progressed to the late stage. In contrast, tumor-promoting Arg-1^+^ macrophages were substantially increased in the tumor stages as shown by subclusters Mφ_c3, c8, and c9 (Figure 3h and i). These results indicate that there was a dynamic picture of tumor-associated macrophage repolarization in PDAC.

### Arginase-1^+^ macrophages and MDSCs may be responsible for immunosuppression in PDAC

To further verify the precise role of the different types of immune cells in PDAC, we performed a correlation analysis based on all of the KPC mice involved in the current study. We combined several subclusters with similar features. For instance, the CD8_c6, c11, and c13 subclusters were recognized as CD8^+^ effector T cells and the CD4_c5 subcluster was identified as CD4^+^ effector T cells (Figure 2d and e). Consistent with the previous results, Arg-1^+^ Macrophages (Mφ_c3, c4, c8, and c9), CD43^+^IgD^−^ naïve B cells (B cell_c2 and c5-7), MDSCs and Tregs were negatively correlated with effector T cells (Teffs), whereas MHC-II^+^/BST2^+^ Macrophages (Mφ_c1, c5, c6, and c10-14), mature B cells and conventional DCs (cDCs, including DC_c6 and c7) were positively correlated with Teffs (Figure S3–e). These correlation analyses further supported that MHC-II^+^/BST2^+^ Macrophages, CD43^−^IgD^+^ mature B cells, and cDCs might contribute to anti-tumor immunity, whereas Arg-1^+^ Macrophages, CD43^+^IgD^−^ immature B cells, MDSCs, and Tregs may have immunosuppressive effects in PDAC. We also performed a correlation analysis on the immunotype of each mouse based on Pearson's correlation coefficient. Among the 22 mouse samples, we detected 100 pairs of samples that showed strong correlation with each other. Specifically, individuals within the normal and ADM stages were immunotypically well-correlated with each other (36 pairs upper left), as were individuals within PanIN and early-stage tumors (36 pairs lower right). 3 individuals correlated with both Normal/ADM groups and PanIN/ES Tumor gourps (highlighted with orange line), indicating that they may be in a transitional state. However, individuals within late-stage tumors were complicated and showed little similarity with each other (only 1 pair showed strong correlation), indicating the particularity and complexity of the immune microenvironment in late-stage PDAC (Figure S3f).

### Recapitulation of the immune landscape in human PDAC

**Figure 4:**
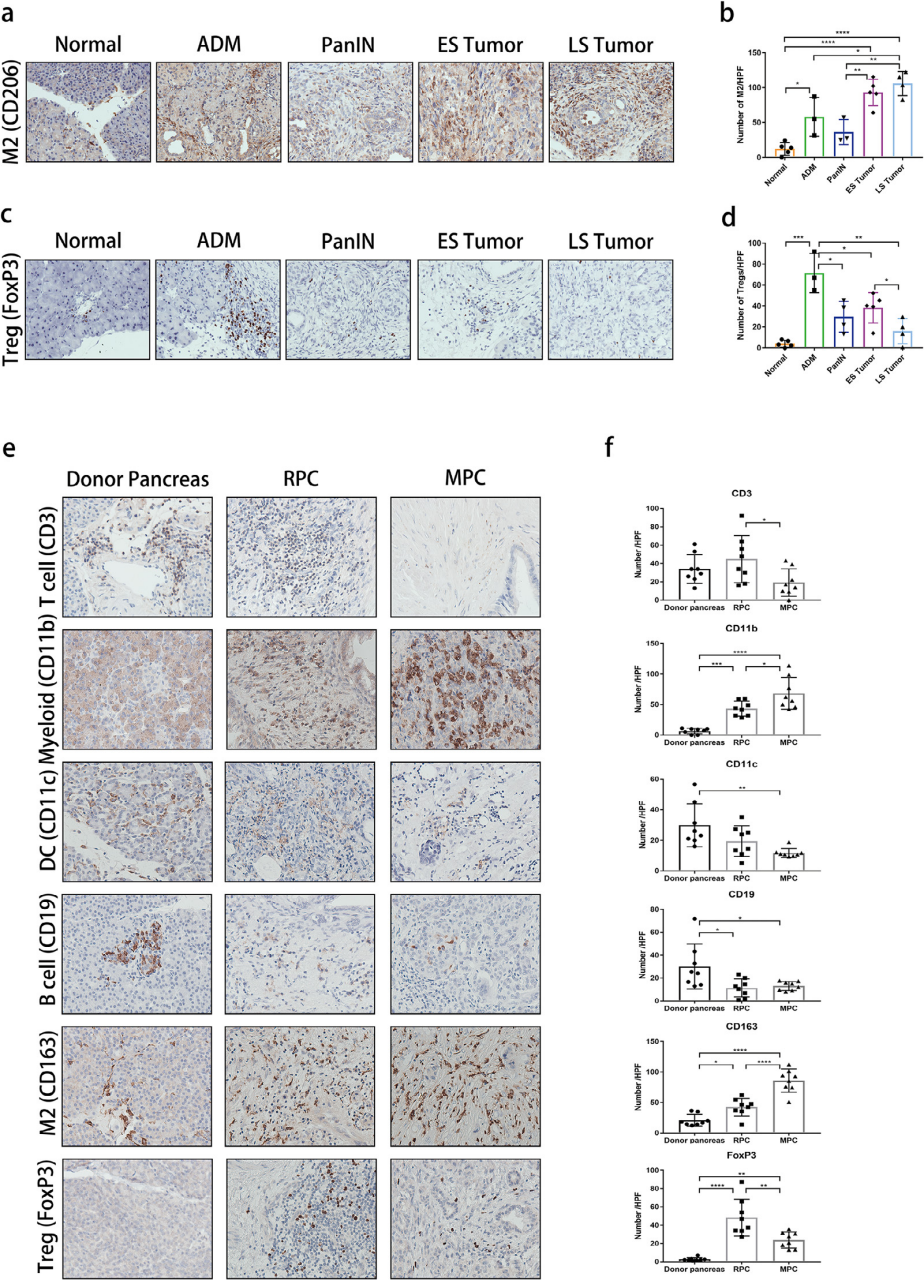
Verification of the immunotype in mouse and patient samples by IHC staining. (a, c) Representative IHC images of CD206+ M2 macrophages (a) and FoxP3+ Tregs (c) in mouse PDAC samples (400×). (b, d) Statistics of the number of M2 macrophages (b) and Tregs (d) in a 400× field, each dot represents the mean of three random field in one sample. (e) Representative IHC images of CD3, CD11b, CD11c, CD19, CD163 and FoxP3 staining using human PDAC sample (400×, *n* = 8 in each group). (f) Statistics of the number of T cells, myeloid cells, DCs, B cells, M2 macrophages and Tregs in a 400× field, each dot represents the mean of three random field in one sample. *n* = 8 in each group. Donor pancreas: pancreas from organ donors, RPC: resectable pancreatic cancer, MPC: metastatic pancreatic cancer. For (b), (d) and (f), bar indicates mean ± SD, * *p* < 0.05, ** *p* < 0.01, *** *p* < 0.001, one-way ANOVA, multiple comparisons.

To investigate whether human PDAC manifests in similar dynamic changes of the immune microenvironment with mouse PDAC, we tested key markers for the detection of certain types of immune cells in mouse and human FFPE samples using immunohistochemistry. CD206 and FoxP3 were used to detect M2-like macrophages, which were equivalent to Arg-1^+^ macrophages^27^ and Tregs,^28^ respectively. Immunohistochemistry staining showed the significant accumulation of CD206^+^ macrophages in both early- and late-stage tumors, which resembled that of Arg-1^+^ macrophages detected by CyTOF (Figure 4a and b). As expected, immunohistochemistry confirmed that Tregs were exclusively abundant in the ADM stage (Figure 4c and d). Consistently, T cells, B cells, and DCs were rarely present in metastatic pancreatic cancer, whereas the immunosuppressive cells, including MDSCs (CD11b) and M2-like macrophages (CD163), increased from the donor pancreas to metastatic PDAC (Figure 4e and f). The abundance of Tregs in metastatic PDAC samples decreased to low levels, which was consistent with the findings from the KPC model. These data show that the temporal dynamic changes to the main types of immune cells detected by CyTOF and immunohistochemistry were largely similar. Moreover, these results suggest that KPC mouse-derived spontaneous PDAC could reliably recapitulate both the histopathological and immunological features of human PDAC.

### Early removal of PD-L1 reverses immunosuppression in PDAC and prolongs long-term survival of mice

**Figure 5:**
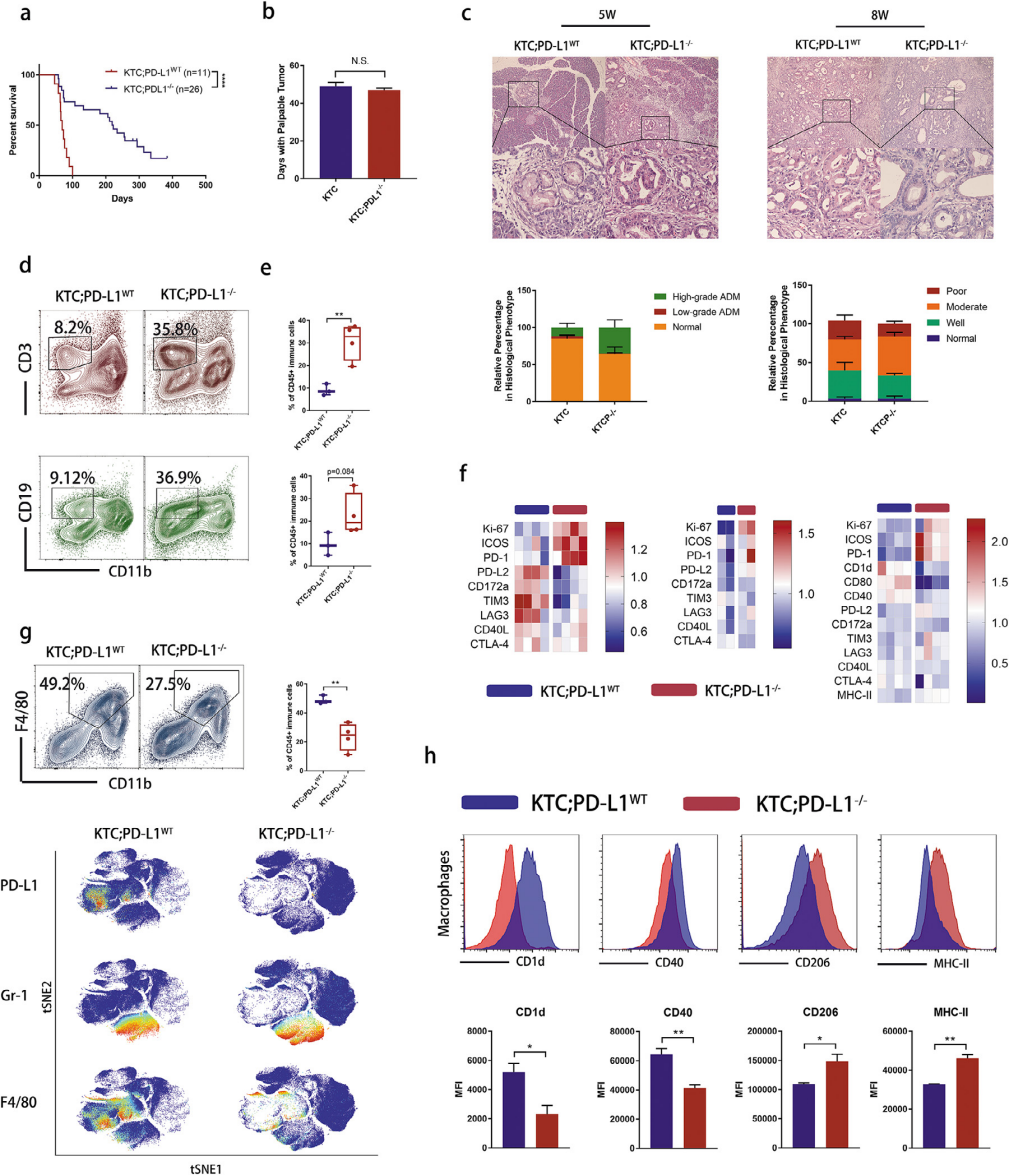
Early intervention by PD-L1 knockout activates tumor immunity and prolonges mouse survival. (a) Mouse survival rates are shown, * *p* < 0.05, ** *p* < 0.01, Log-rank test. (b) Days for mice to develop a palpable pancreas tumor. N.S., no significance. (c) Upper: representative images of hematoxylin-eosin (HE) staining for 5-week precancerous pancreas and 8-week tumor tissues. Lower: quantification for different morphologies (*n* = 4 for KTC and *n* = 5 for KTC;PD-L1^−/–^). (d) Representative flow cytometry dot plots of T cell and B cell in KTC;PD-L1^WT^ and KTC;PD-L1^−/−^ mice. (e) Statistics of percentage of T and B cell (*n* = 3 for KTC and *n* = 4 for KTC;PD-L1^−/–^), ** *p* < 0.01, student *t* test. (f) Heatmap of CD4^+^ T cell, CD8^+^ T cell and B cell clusters in CyTOF analysis, Each column refers to one cluster and each row refers to one marker. (g) Upper left: representative flow cytometry dot plots of macrophages; Upper right: corresponding statistics, ** *p* < 0.01, student *t* test; Lower: tSNE plots of macrophages in each group. (h) Upper: representative flow cytometry histogram of TAM-expressed CD1d, CD40, CD206 and MHC-II. Lower: statistics of mean fluorescence intensity, * *p* < 0.05, ** *p* < 0.01, student *t* test.

Given that the immunosuppression of PDAC was aggravated progressively, we proposed that early immune interference may lead to greater benefit. To get follow-up data over a shorter period of time, we crossed Kras^G12D/+^;Tgfbr2^flox/flox^;Ptf1a-Cre (KTC) mice, which would develop PDAC with 100% penetrance and a median survival of 59 days,^14^ with PD-L1 knockout mice (KTC;PD-L1^−/−^ mice) to observe whether the immunosuppression in PDAC can be reversed by germline knockout of PD-L1. Both KTC and KTC;PD-L1^−/−^ mice developed palpable PDAC at about six weeks of age indistinguishably (Figure 5b). However, KTC;PD-L1^−/−^ mice showed much longer survival than KTC mice (Figure 5a). Imaging of precancerous (5 weeks old) and tumoral (8 weeks old) tissues showed that, knockout of PD-L1 did not prevent tumorigenesis (Figure 5c). Flow cytometry and CyTOF analysis showed more tumor-infiltrating lymphocytes in 8-week PD-L1 knockout tumors (Figures 5d–e and S4,4b). CD4^+^/CD8^+^ T cell and B cell populations expressed high levels of Ki-67, ICOS, PD-1 and low levels of PD-L2, TIM3, LAG3 and CTLA-4, suggesting a proliferative and active phenotype (Figure 5f). To our surprise, PD-L1 was expressed exclusively in tumor-associated macrophages (TAMs, Figure 5g). Knocking out PD-L1 could cause these TAMs to almost disappear (Figures 5g and S4c). However, the remaining TAMs in KTC;PD-L1^−/-^ tumors was more like an M2-like phenotype with higher expression of CD206 and Arg-1 and lower expression of stimulatory CD40 and CD1d (Figures 5h and S4d). Altogether, these data suggest that PD-L1 may play an immunosuppressive role only in established tumors. Early interference of PD-1/PD-L1 interaction triggers anti-tumor immunity in PDAC, but is insufficient to reverse the immunosuppressive phenotype of the remaining TAMs.

### Arginase-1 inhibitor synergizes with anti-PD-1 antibody to enhance anti-tumor immunity

**Figure 6:**
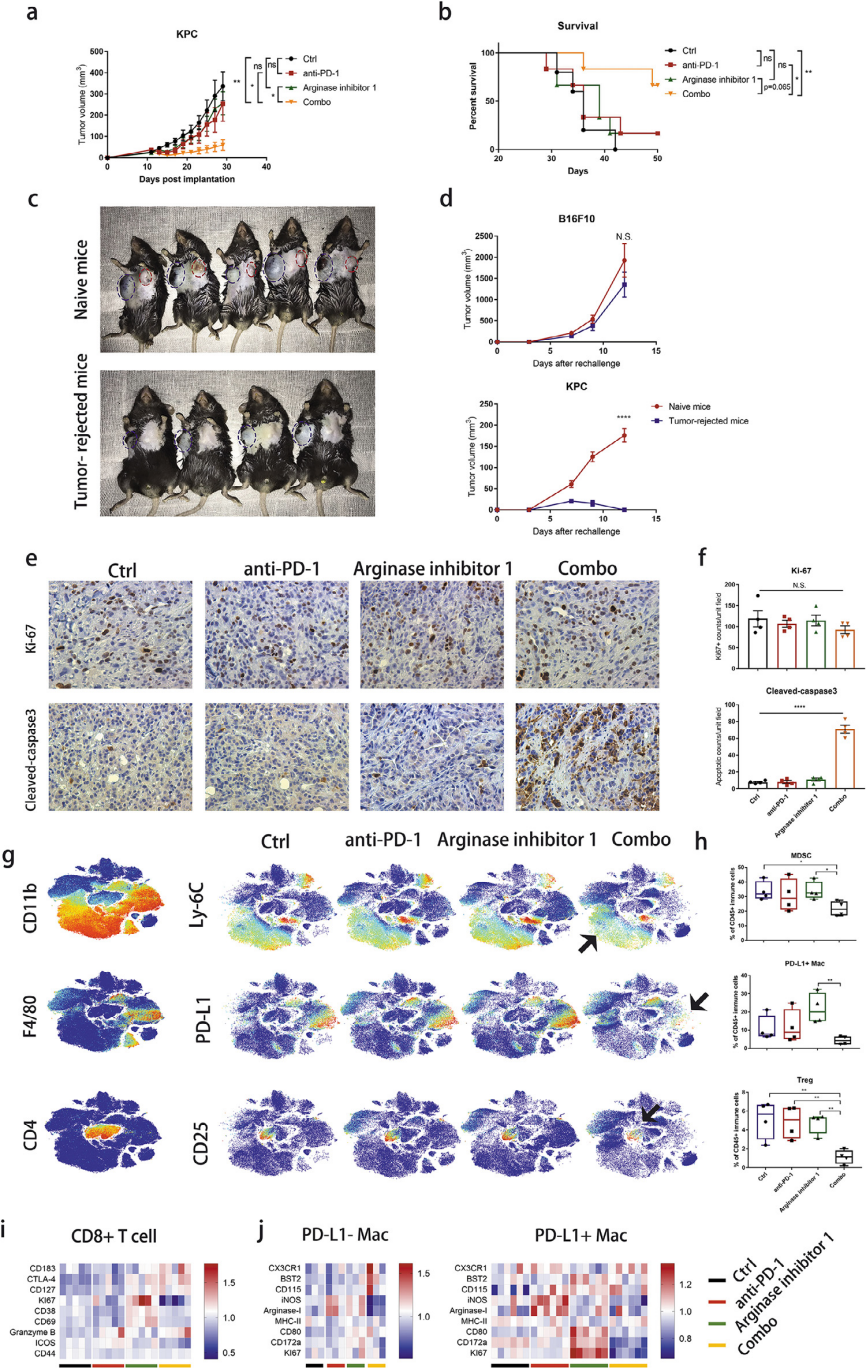
Arginase-1 inhibitor synergizes anti-PD-1 antibody therapy. (a) Tumor growth curve of KPC syngeneic model is shown as mean±SEM of 6,7 mice per group. * *p* < 0.05, ** *p* < 0.01. (b) Mouse survival rates are shown, * *p* < 0.05, ** *p* < 0.01, Log-rank test. (c) Mice engrafted with KPC tumor cells were treated with anti-PD-1 mAb w/o Arg-1inhibitor combination therapy, 1 mouse from anti-PD-1 mAb treated group and 3 mice from combined group achieved tumor regression. After 2 months, the KPC survived mice were rechallenged subcutaneously with B16F10 (right flank) and KPC (left flank) tumors. Naïve mice were also inoculated in the same manner as control. (d) Rechallenged tumor size were measured and are shown as mean±SEM. N.S. no significance, **** *p* < 0.0001, student *t* tests. (e) Representative IHC images of Ki-67 and Cleaved-caspase3 in each group (400×). (f) Statistics of the number of Ki-67^+^ and Cleaved-caspase3^+^ cells in a 400× field. (g) tSNE plots of intratumoral immune cells in different groups. CD11b and Ly-6C were used as markers of MDSC; F4/80 and PD-L1 were used to discribe PD-L1^+^ macrophage; CD4 and CD25 represent Treg; (h) Boxplots shows statistics of corresponding immune cell types. * *p* < 0.05, ** *p* < 0.01, student *t* tests. (i, j) Heatmap of CD8^+^ T cell clusters, PD-L1^−^ and PD-L1^+^ macrophage clusters. Each column refers to one cluster and each row refers to one marker. Red means high expression while blue means low expression. Mac: Macrophage.

We speculated that combined treatment with myeloid cell-targeting agents and anti-PD-1 antibodies could improve the therapeutic efficacy even in late tumor stage. Since M2-like macrophage that showed high expression of Arg-1 was one of the major immunosuppressive cell types in PDAC, we thus combined a small molecule compound Arg-1 inhibitor (PubChem CID 66833213) with anti-PD-1 antibody to treat KPC and PancO2 tumor-bearing mice. The combined therapy significantly inhibited KPC and PancO2 tumor growth (Figures 6a and S5a) without significant toxicity (Figure S5b), resulting in prolonged survival compared with other groups (Figure 6b). Single neither Arg-1 inhibitor nor anti-PD-1 antibody achieved obvious tumor growth inhibition. In addition, combined therapy achieved KPC syngeneic tumor rejection in three mice and anti-PD-1 antibody monotherapy achieved in one mouse. To confirm the long-term specific anti-tumor immune memory induced by immunotherapy, the survived mice were rechallenged with double doses of KPC cells and mouse syngeneic melanoma cells B16F10 as control. Indeed, all the survived mice were resistant to rechallenge with KPC cells but not B16F10 cells, while both KPC and B16F10 showed apparent tumor growth in naïve control mice (Figure 6c and d). IHC staining of Ki-67 and cleaved-caspase3 showed that tumor cell proliferation was not inhibited in no treatment group, while combination therapy significantly induced tumor cell apoptosis compared to either single treatment (Figure 6e and f), suggesting that anti-PD-1 mAb and Arg-1 inhibitor have no direct proliferative toxicity and neither single treatment was sufficient to activate the immune killing to tumor cells. Immune microenvironment profiling with CyTOF confirmed that combined therapy significantly decreased tumor-infiltrating MDSCs, PD-L1^+^ macrophages and Tregs (Figure 6g and h). CD3^+^CD49b^+^ NKT cells and IgD^+^CD19^+^ B cells seemed increased although statistically insignificant (Figure S5c and d). However, the fraction of both CD4^+^/CD8^+^ T cells were not changed (Figure S5e and f). We then studied the phenotype of both CD8^+^ T cells and non-Treg CD4^+^ T cells. CD4^+^/CD8^+^ T cells in the combined therapy expressed relatively high level of CD183 (also known as CXCR3) and CD127, suggesting an enhanced recruitment and memory feature (Figures 6i and S5g). CD8+ T cells also highly expressed granzyme B in anti-PD-1 and combo group, showing cytotoxic activity (Figure 6i). We also observed enhanced expression of CTLA-4 on CD8^+^ T cells, suggesting an immune checkpoint compensation mechanism that restricted CD8^+^ T cell expansion. In addition, the expression of iNOS and Arg-1 was downregulated while the levels of CX3CR1, BST2, and CD115 were upregulated on both PD-L1^−^ and PD-L1^+^ macrophages (Figure 6j), indicating a pro-inflammatory phenotype. Thus, combination of Arg-1 inhibitor and anti-PD-1 antibody showed an inspiring treatment efficacy by reversing myeloid cell-derived immunosuppression.

## Discussion

Under conditions of oncogenic mutation, sustained injury and environmental factors, patients can develop chronic pancreatitis resulting in infiltration of immune cells, desmoplasia and emergence of ADM.^18^ The immune cells, capillaries, activated fibroblasts and extracellular matrix (ECM) form the microenvironment of the tumor-initiating pancreas.^29^ During the transformation from normal pancreas to ADM to PanIN and to PDAC, the desmoplastic reaction keeps increasing and is a cardinal feature of pancreatic cancer.^11^^,^^30^ There is a dynamic interaction between metaplastic/cancer cells and surrounding stroma. The metaplastic/cancer cells keep developing autonomous defects and secrete a range of cytokines (e.g. IL-1α, IL-18, TNF-α, TGF-β et al.), to alter immune cell phenotype and to support the activation and proliferation of fibroblasts.^31^^,^^32^ Concurrently, fibroblasts may adopt a secretory phenotype, producing ECM protein, enzymes, cytokines and chemokines. The secretome of fibroblasts may dramatically evolve during tumor initiation and progression, thus potentially affecting tumor immunity differently at different stages.^29^^,^^33^, ^34^, ^35^, ^36^

Since both tumor and immune cells co-evolve with tumor progression, investigating the dynamic immune landscape during tumor development is important for understanding the biological stages of the disease. Additionally, identifying the current immune microenvironment of the tumor is particularly essential for selecting the optimal immunotherapy. However, it is impractical and non-ethical to repeatedly obtain samples from patients. In addition, it is impossible to acquire ADM and PanIN pancreatic samples from humans. To explore the dynamic immune landscape of PDAC, we took advantage of the KPC mouse model, which has been proven to faithfully mimic the features of humans in PDAC studies. Similar to human PDAC, KPC mouse PDAC is also triggered by mutant *Kras* and *Trp53*, and exhibits an extremely similar progression pathway from ADM, PanIN to invasive tumors and ultimately leads to death with or without metastasis.^17^ Therefore, the KPC mouse model represents an alternative method of studying the dynamic changes to local immunity in PDAC. In this study, we used KPC mice to identify the dynamic changes in the temporal immunotype for the entire process of tumor initiation and progression (Figure S6). These findings were then recapitulated in human patients at different stages.

Although PDAC has been described as an immunologically-cold tumor with limited immune cell infiltration,^37^ it is normally derived from a static sampling. In our study, we revealed the dynamic changes in the immunotype along with histopathological evolvement in PDAC. Most importantly, we identified two stages of immunosuppression mediated by distinct immune cells during PDAC development. The accumulation of Tregs and absence of Teffs in the ADM stage suggests that compromised adaptive immunity is primarily responsible for facilitating the initiation of PDAC. However, in metastatic PDAC when the tumor is extensively evolved, MDSCs and Arg-1^+^ M2-like macrophages represent the main contributors of immunosuppression, which suggests impaired innate immunity at this stage. The difference between the two stages highlights the importance of correcting innate immunity in PDAC. Moreover, treatment with immune checkpoint inhibitors failed to show any efficacy in PDAC. Previous studies demonstrated that a low abundance of T cells was the primary reason for this effect^38^; however, our study suggests that impaired innate immunity (e.g., phagocytosis and antigen-presenting capacity) are also important factors. We proposed that targeting Arg-1 could synergize immune checkpoint inhibition (ICI) therapy. Considering the high consistency of immune microenvironment between PDAC of KPC mice and human, targeting Arg-1 may also be a promising strategy to improve anti-PD-1 antibody therapy and increase overall survival in human patients.

Tumors with an immune-devoid phenotype characterized by low T and B cell infiltration have been found to indicate a worse prognosis.^39^^,^^40^ According to our findings, PDAC with an immune-devoid phenotype may be due to a late stage rather than a special subtype of PDAC. In the early stage of pancreatic cancer, although there was a gradual accumulation of MDSCs and TAMs, the effector T and B cells remained abundant. This finding suggests that immunotherapy is more likely to be successful during the earlier stages of PDAC. It is difficult to track whether KPC or KTC mice have metastasized, we generated PD-L1-knockout KTC mice, although not optimal, as an early intervention model, and showed successful immune activation and survival extending.

Our study provides insight into several key components of intratumoral immune cells. To our knowledge, this is the first report demonstrating that BST2^+^ macrophages may participate in the reprogramming of the tumor microenvironment in PDAC. BST2, also known as tetherin or CD317, is an interferon-induced protein that inhibits the release of viral particles from infected macrophages.^41^ BST2 has also been reported to have a pro-inflammatory function through the Syk/TRAF2/TRAF6/TAK1/NF-κB pathway under conditions of viral infection.^42^ However, BST2 has rarely been studied in cancer. In the present study, macrophages exhibited either an antigen-presenting/pro-inflammatory (MHC-II^+^/BST2^+^ macrophage) or immunosuppressive function (Arg-1^+^ macrophages). The cellular fate of Ly-6C^+^ monocytes changes from pro-inflammatory to immunosuppressive macrophages over time as PDAC progresses. This suggests that BST2 expression may represent a marker of anti-tumor macrophages in the PDAC microenvironment. Thus, redirecting monocyte/macrophage differentiation to the anti-tumoral branch may be a promising method of stimulating anti-tumor immunity. However, further studies on the induction and function of BST2 in macrophages are warranted to expand our understanding of the precise role of such macrophage subsets.

We also observed that PDAC displayed a prominent presence of B cells, which are crucial for adaptive immunity. B cells have been reported to be either immunostimulatory^43^ or immunosuppressive.^44^^,^^45^ The controversial role of tumor-infiltrating B cells has led to differing opinions about whether immunotherapies should be designed to enhance or limit these cells. A recent study reviewed 69 studies involving 19 types of cancers and showed that approximately 50% of studies reported a positive prognostic effect for B cells, whereas the remaining studies found a neutral (40.7%) or negative (9.3%) effect.^46^ Recently, several studies showed that tertiary lymphoid structures and B cell facilitate immunotherapeutic responses in melanoma, sarcoma and breast cancer.^47^, ^48^, ^49^, ^50^ Here, we showed that a major group of B cells with a CD43^−^IgD^+^ expression pattern was excluded in the immunosuppressive stages, concordant with CD4^+^ and CD8^+^ effector T cells, whereas the opposite was observed in the CD43^+^ IgD^−^ B cell group. When the immunosuppression of tumor microenvironment was reversed either by PD-L1 depletion or Arg-1-targeted ICI combined therapy, B cells showed an obvious increase. Thus, we postulated that CD43^−^IgD^+^ mature B cells may contribute to anti-tumor immunity and may promote immunotherapy in early pancreatic cancer. However, the precise role of B cells in PDAC remains largely unknown. Future studies are required to further classify B cells and elucidate their precise functions.

There are some limitations associated with the current research. First, our sampling strategy cannot fully recapitulate the actual evolvement of local immunity in PDAC. Second, PanIN was not further divided into PanIN1, PanIN2, and PanIN3. Third, the number of mice in each group was relatively low. In addition, the timing of tumor initiation in KPC mice can vary slightly because it is dependent on the random loss of another *Trp53* allele. The difference in the timing and pattern of the *Trp53* mutation may influence the tumor cell biology and be associated with a variable immune landscape. Furthermore, since no human pancreatic ADM and PanIN samples can be obtained, and immunohistochemistry is methodologically weak for the accurate differentiation of certain cell types, we can only infer dynamic changes in the immune microenvironment from the mouse model.

## Contributors

Liang T and Zhang Q conceived the study; Yang J, Wang J, Hong Z, Wang J collected the samples; Wei S and Sun K analyzed the pathology; Yang J, Zhang Q, Lou Y, Chen Y, Sheng J and Su W analyzed the data; Yang J and Zhang Q drafted the manuscript. Liang T and Bai X supervised the study. All authors revised the manuscript and approved the final version of the manuscript.

### Data sharing

The datasets used and/or analysed during the current study are available from the corresponding authors on reasonable request.

## Declaration of interests

The authors declare no potential conflicts of interest.

## Appendix Supplementary materials

Image, application 1
