# Supplementary material for: Dynamic profiling of immune microenvironment during pancreatic cancer development suggests early intervention and combination strategy of immunotherapy
Source: eBioMedicine. 2022 Mar 19;78:103958. doi: 10.1016/j.ebiom.2022.103958 (PMC8943259; doi:10.1016/j.ebiom.2022.103958)
Supplement: Supplementary file 1 [file mmc1.docx]

# Supplementary Materials for

Leveraging immunotherapy to treat pancreatic cancer based on the features of immunosuppression in different stages

Yang Jiaqi, Zhang Qi, Wang Junli, Lou Yu, Hong Zhengtao, Wei Shumei, Sun Ke, Wang Jianing, Chen Yiwen, Sheng Jianpeng, Su Wei, Bai Xueli, Liang Tingbo

Correspondence to: liangtingbo@zju.edu.cn or shirleybai@zju.edu.cn

**This file includes:**

Figures S1 to S6

Tables S1 to S6


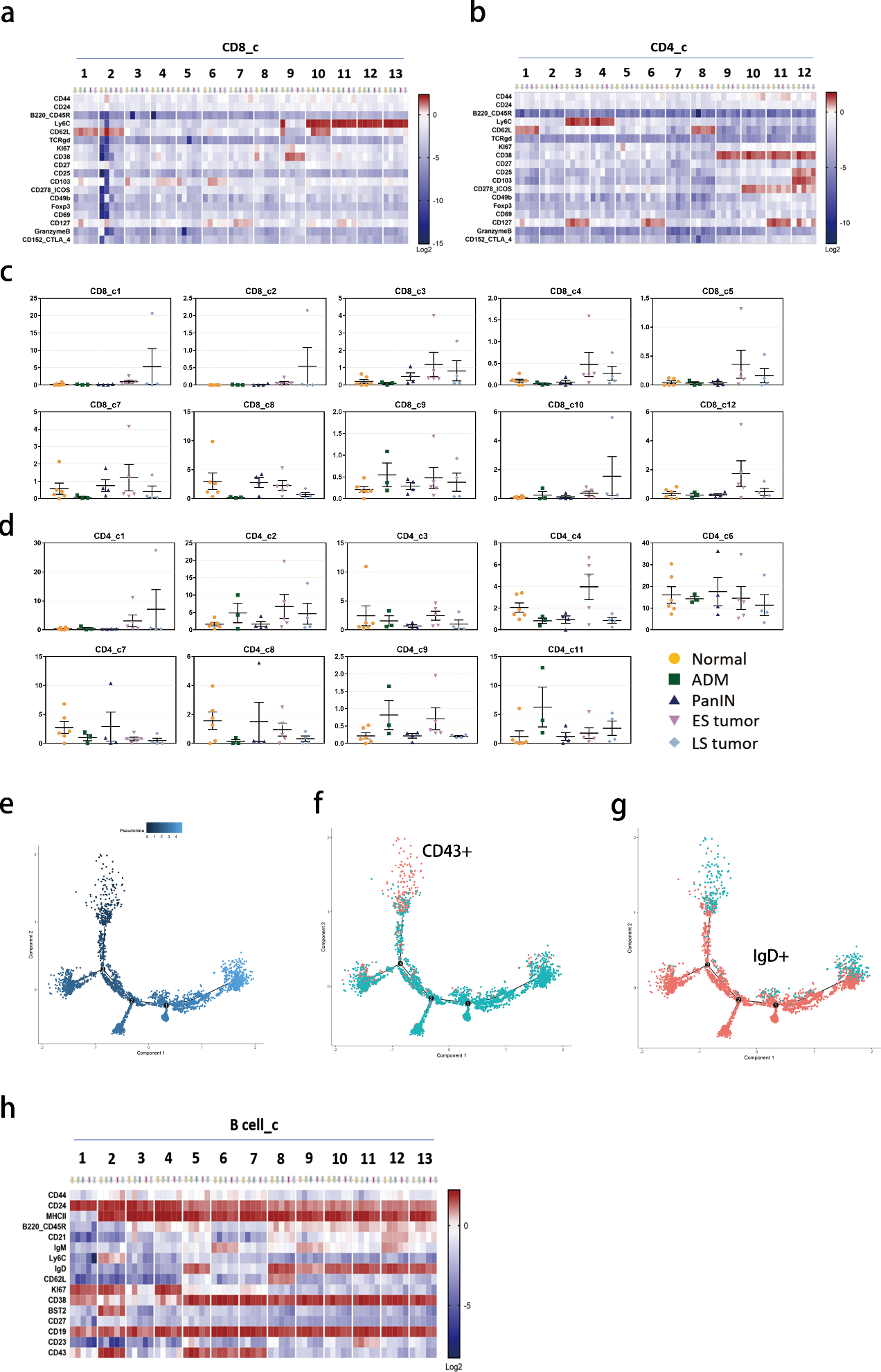


Figure. S1.

Phenotypes of lymphoid cells. (a, b, h) Heatmap of CD8+ T cell, CD4+ T cell and B cell clusters with normalized expression of selected markers. (c, d) Percentage of CD8+ and CD4+ T cells in clusters without significant change during PDAC development (mean percent ± SD of total T cells). (e-g) CD43+ and IgD+ B cell distribution on pseudotime trajectory of B cell development.


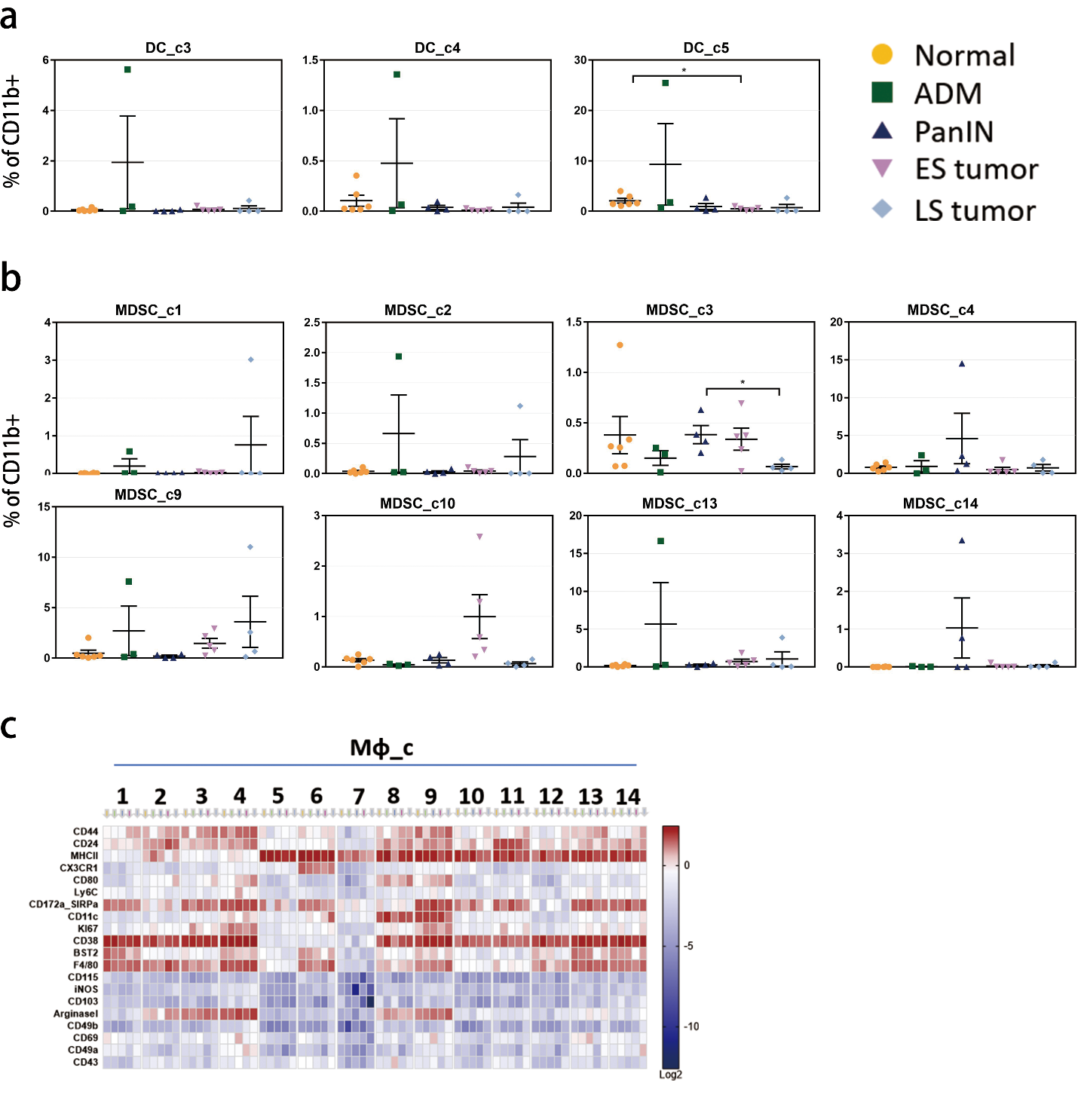


Figure. S2.

**Reclustering of myeloid cells.** **(a, b)** Percentage of DCs and MDSCs in clusters without significant change during PDAC development (mean percent ± SD of total T cells). **(c)** Heatmap of m clusters with normalized expression macrophage clusters of selected markers.


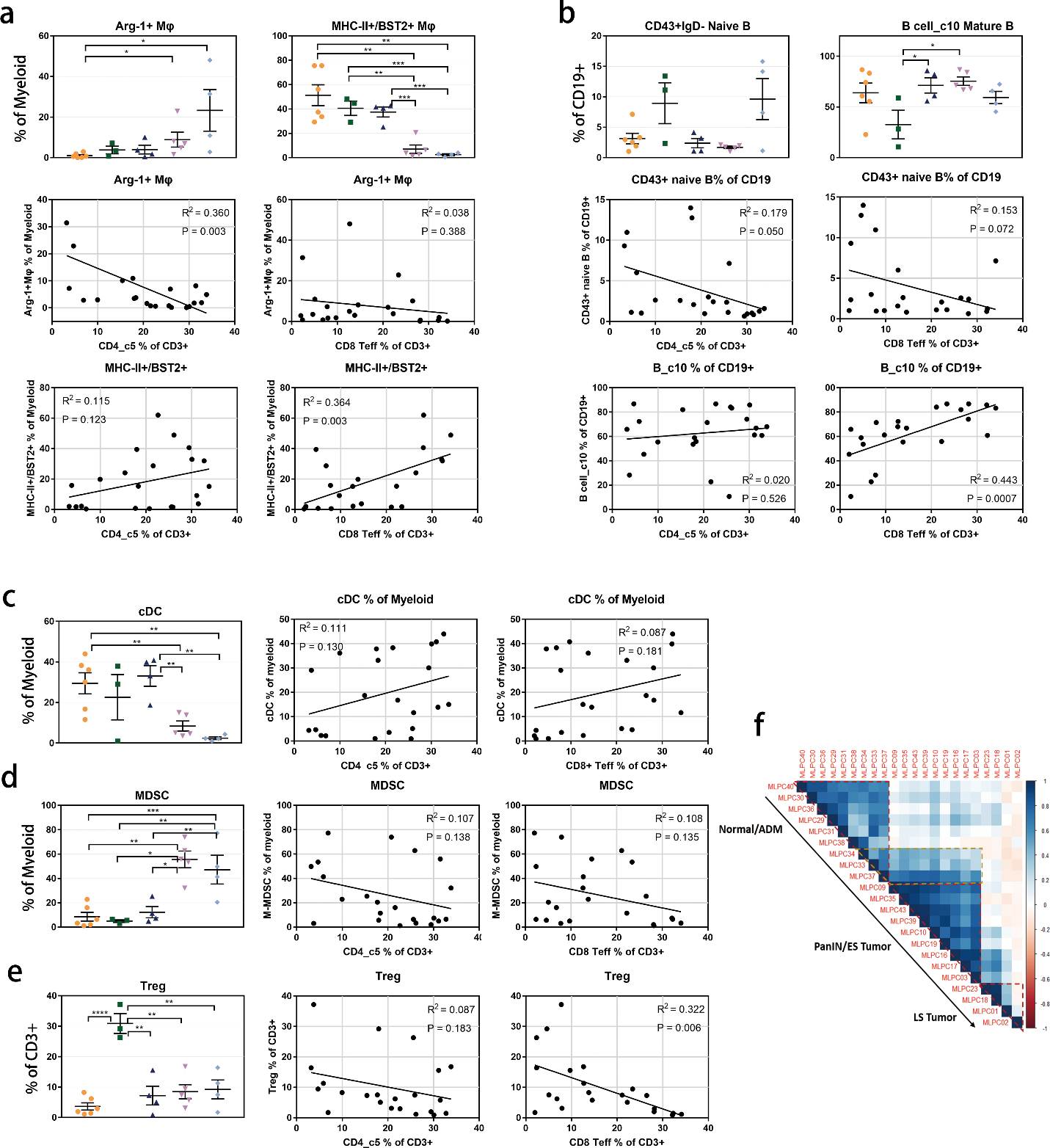


**Figure. S3.**

**Correlation analysis between intratumoral immune cell populations and individuals.** **(a)** Evolvement of Arg-1+ and BST2+/MHC-II+ macrophages for each developmental stage and their correlationship with CD4_c5 cluster or CD8+ Teff. Arg-1+ macrophage includes clusters of Mφ_c3, c4, c8 and c9. BST2+/MHC-II+ macrophage includes clusters of Mφ_c1, c5, c6, and c10-c14. CD8+ Teff includes clusters of CD8_c6, c11 and c13. **(b)** Evolvement of CD43+IgD--naive and CD43-IgD+-mature B cells for each developmental stage and their correlationship with CD4_c5 cluster or CD8+ Teff. CD43+IgD--naive B cell includes clusters of B cell_c2 and c5-7. CD43-IgD+-mature B cell refers to B cell_c10. **(c-e)** Evolvement of conventional DC (cDC), MDSC and Treg for each developmental stage and their correlationship with CD4_c5 cluster or CD8+ Teff. cDC includes clusters of DC_c6 and c7. MDSC includes clusters of MDSC_c5-c14. Treg includes clusters of CD4_c10-c12. **(f)** Correlation between different individuals, the individual order has been manually adjusted to mimic the developmental process. For scatter plots, bar indicates mean percent ± SD of total myeloid cells (a, c and d), total B cells (b) or total T cells (e), * p<0.05, ** p<0.01, *** p<0.001, one-way ANOVA, multiple comparisons.


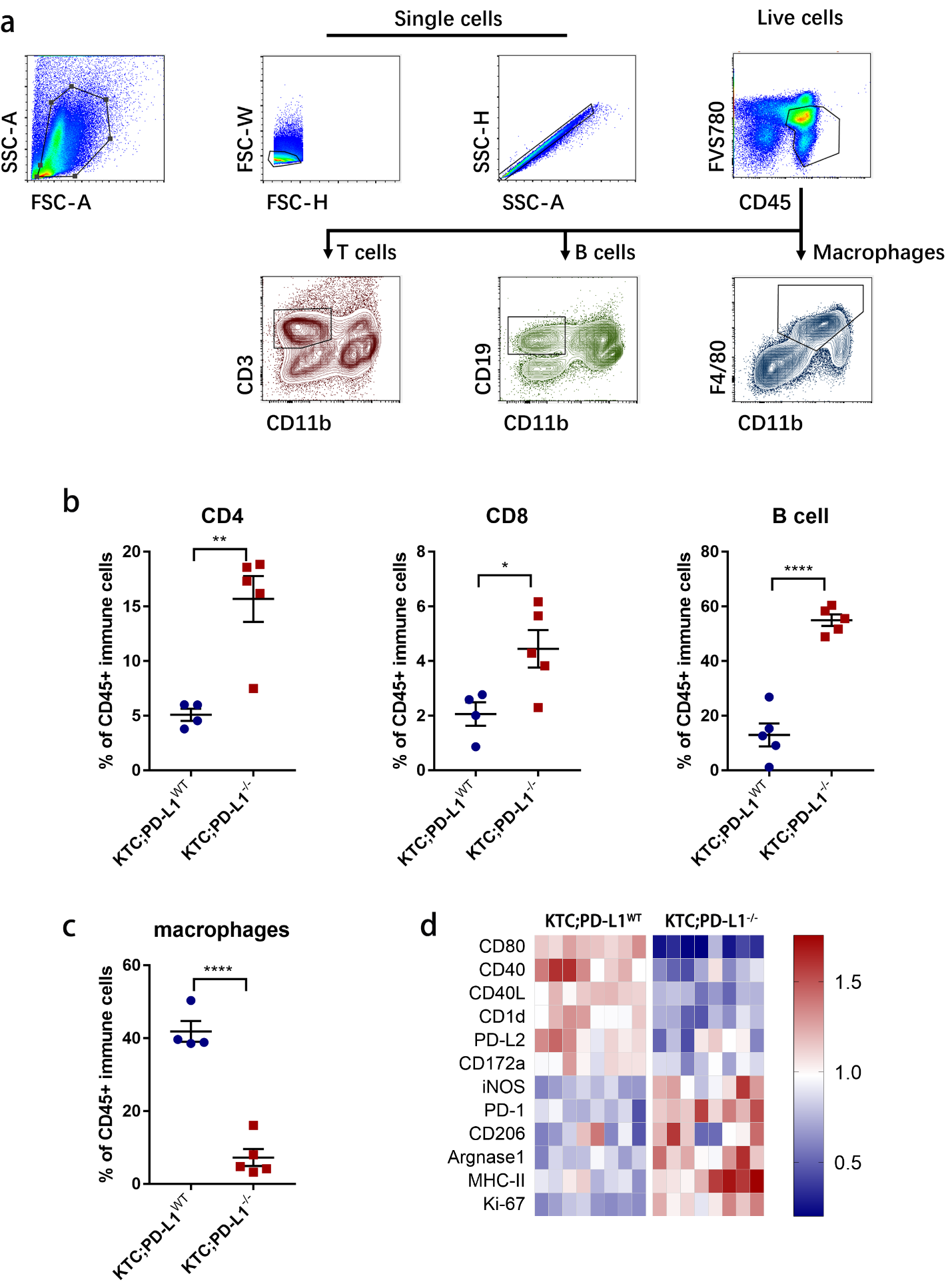


**Figure. S4.**

**(a)** Gate strategy to rule out the adhesions and dead cells. **(b)** Statistics of CD4^+^ T cells, CD8^+^ T cells and B cells detected by CyTOF analysis in PD-L1-wildtype (n=4) and -knockout (n=5) KTC mice tumors. * p<0.05, ** p<0.01, **** p<0.0001. **(c)** Statistics of macrophages in PD-L1-wildtype (n=4) and -knockout (n=5) KTC mice tumors. **** p<0.0001. **(d)** Heatmap of macrophage clusters in CyTOF analysis. Each column refers to one cluster and each row refers to one marker. Red means high expression while blue means low expression.


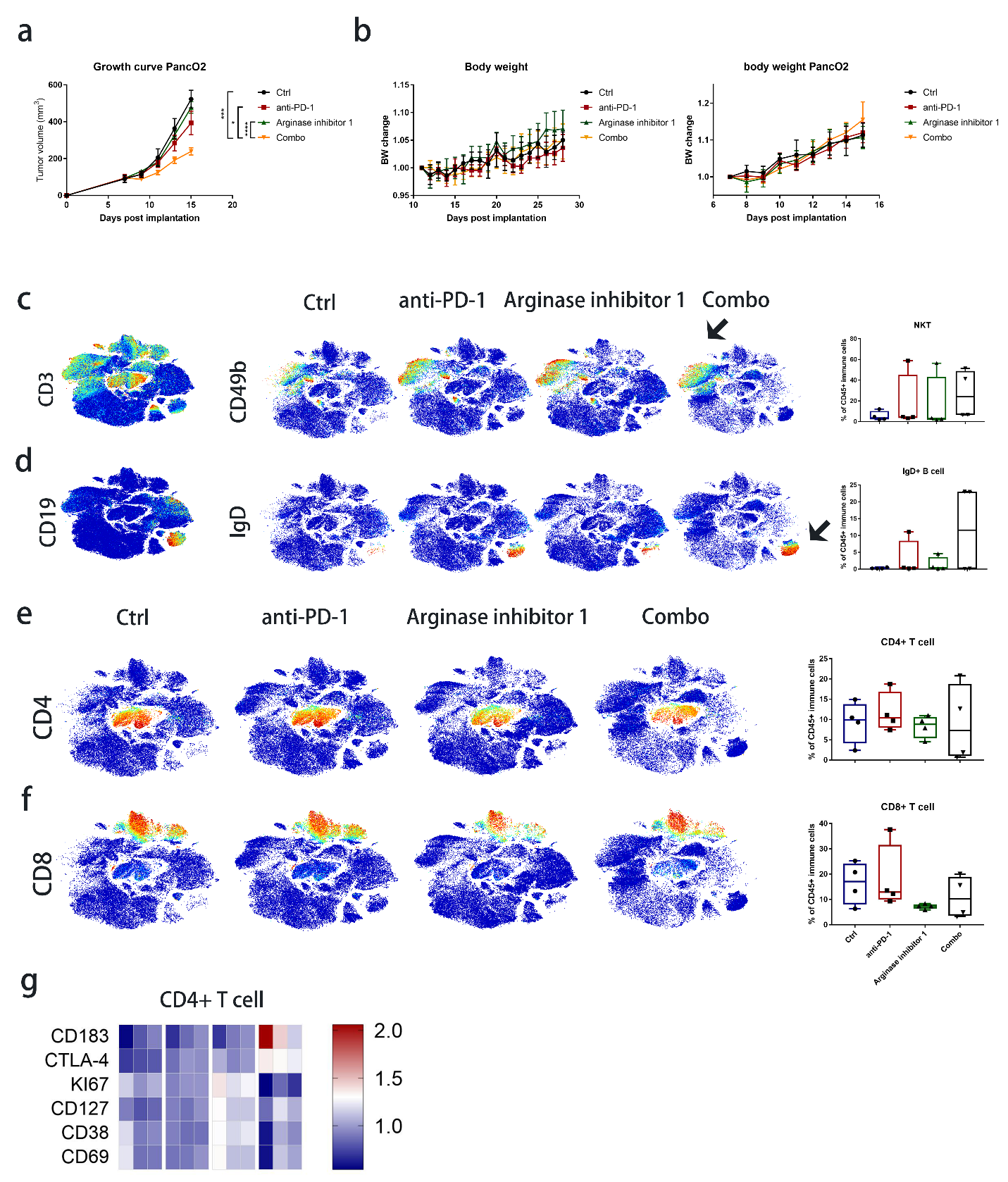


**Figure. S5.**

**(a)** Tumor growth curve of PancO2 syngeneic model is shown as mean±SEM of 6-7 mice per group. * p<0.05, *** p<0.001, **** p<0.0001. **(b)** Body weight changes of KPC and PancO2 engrafted mice are shown as mean±SEM, 6-7 mice per group. tSNE plots of intratumoral immune cells in different groups. **(c-f)** tSNE plots of intratumoral immune cells in different groups. CD3 and CD49b were used as markers of natural killer cell (NKT) (c); CD19 and IgD were used to discribe IgD^+^ B cell (d); CD4 and CD8 represent CD4^+^ T cell and CD8^+^ T cell respectively (e, f). Statistics of corresponding immune cell types are listed in the right side. Boxplots with all points are shown. **(g)** Heatmap of CD4+ T cell clusters. Each column refers to one cluster and each row refers to one marker. Red means high expression while blue means low expression.


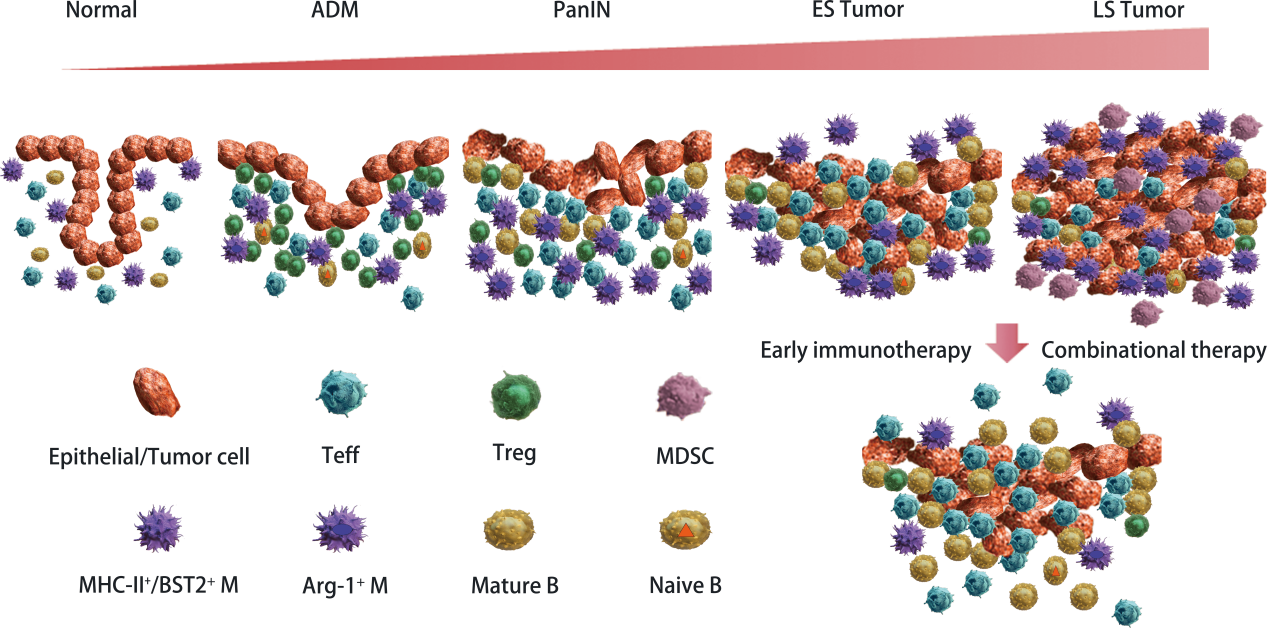


**Figure. S6.**

Graphical abstract of this study. The immune microenvironment of PDAC changes dynamically along with tumor initiation and progression. Immunotherapy is more likely to be effective in treating early-stage tumor. For late-stage PDAC, arginase-1 inhibitor and anti-PD-1 combined immunotherapy maybe a promising strategy.

| No. | Channel | Antibody | Clone | No. | Channel | Antibody | Clone | |
| --- | --- | --- | --- | --- | --- | --- | --- | --- |
| 1 | 89Y | CD45 | 30-F11 | 22 | 159Tb | F4/80 | C1:A3-1 | |
| 2 | 115In | CD3e | 145.2C11 | 23 | 160Gd | CSF-1R | AFS98 | |
| 3 | 139La | CD44 | IM7 | 24 | 161Dy | iNOS | CXNFT | |
| 4 | 141Pr | CD24 | M1/69 | 25 | 162Dy | CXCR3 | CXCR3-173 | |
| 5 | 142Nd | MHC II | Y3P | 26 | 163Dy | CD25 | 3C7 | |
| 6 | 143Nd | B220_CD45R | RA3-6B2 | 27 | 164Dy | CD103 | 2E7 | |
| 7 | 144Nd | CX3CR1 | SA011F11 | 28 | 165Ho | ICOS | C398.4A | |
| 8 | 145Nd | CD21 | 7G6 | 29 | 166Er | Arginase I | polyclone | |
| 9 | 146Nd | IgM | RMM-1 | 30 | 167Er | CD49b | DX5 | |
| 10 | 147Sm | CD80 | 16-10A1 | 31 | 168Er | Foxp3 | FJK-16s | |
| 11 | 148Nd | Ly6C | HK1.4 | 32 | 169Tm | CD69 | H1.2F3 | |
| 12 | 149Nd | SIRPα | P84 | 33 | 170Er | CD49a | HMa1 | |
| 13 | 150Nd | IgD | 11-26c.2a | 34 | 171Yb | CD23 | B3B4 | |
| 14 | 151Eu | CD62L | MEL14 | 35 | 172Yb | CD127 | A7R34 | |
| 15 | 152Sm | CD11c | N418 | 36 | 173Yb | Granzyme B | | GB11 |
| 16 | 153Eu | TCRgd | GL3 | 37 | 174Yb | CTLA-4 | UC10-4B9 | |
| 17 | 154Sm | KI67－m/h | SolA15 | 38 | 175Lu | TCRab |  | |
| 18 | 155Gd | CD38 | 90 | 39 | 176Yb | CD43 | S11 | |
| 19 | 156Gd | BST2 | 44E9R | 40 | 197gd | CD4 | RM4-5 | |
| 20 | 157Gd | CD27 | LG.3A10 | 41 | 198pt | CD8a | 53-6.7 | |
| 21 | 158Gd | CD19 | 6D5 | 42 | 209Bi | CD11b | M1/70 | |

Table S1.

CyTOF antibodies for KPC mice.

| No. | Channel | Antibody | Clone | No. | Channel | Antibody | Clone | |
| --- | --- | --- | --- | --- | --- | --- | --- | --- |
| 1 | 89Y | CD45 | 30-F11 | 22 | 159Tb | F4/80 | C1:A3-1 | |
| 2 | 115In | CD3e | 145.2C11 | 23 | 160Gd | CSF-1R | AFS98 | |
| 3 | 139La | CD44 | IM7 | 24 | 161Dy | iNOS | CXNFT | |
| 4 | 141Pr | CD24 | M1/69 | 25 | 162Dy | CXCR3 | CXCR3-173 | |
| 5 | 142Nd | MHC II | Y3P | 26 | 163Dy | CD25 | 3C7 | |
| 6 | 143Nd | B220_CD45R | RA3-6B2 | 27 | 164Dy | CD103 | 2E7 | |
| 7 | 144Nd | CX3CR1 | SA011F11 | 28 | 165Ho | ICOS | C398.4A | |
| 8 | 145Nd | CD21 | 7G6 | 29 | 166Er | Arginase I | polyclone | |
| 9 | 146Nd | IgM | RMM-1 | 30 | 167Er | CD49b | DX5 | |
| 10 | 147Sm | CD80 | 16-10A1 | 31 | 168Er | Foxp3 | FJK-16s | |
| 11 | 148Nd | Ly6C | HK1.4 | 32 | 169Tm | CD69 | H1.2F3 | |
| 12 | 149Nd | SIRPα | P84 | 33 | 170Er | PD-L1 | 10F.9G2 | |
| 13 | 150Nd | IgD | 11-26c.2a | 34 | 171Yb | PD-1 | 29F.1A12 | |
| 14 | 151Eu | CD62L | MEL14 | 35 | 172Yb | CD127 | A7R34 | |
| 15 | 152Sm | CD11c | N418 | 36 | 173Yb | Granzyme B | | GB11 |
| 16 | 153Eu | TCRgd | GL3 | 37 | 174Yb | CTLA-4 | UC10-4B9 | |
| 17 | 154Sm | KI67－m/h | SolA15 | 38 | 175Lu | TCRab |  | |
| 18 | 155Gd | CD38 | 90 | 39 | 176Yb | CD43 | S11 | |
| 19 | 156Gd | BST2 | 44E9R | 40 | 197gd | CD4 | RM4-5 | |
| 20 | 157Gd | CD27 | LG.3A10 | 41 | 198pt | CD8a | 53-6.7 | |
| 21 | 158Gd | CD19 | 6D5 | 42 | 209Bi | CD11b | M1/70 | |

Table S2.

CyTOF antibodies for KPC syngeneic model.

| Cell Types | Symbols | | | | |
| --- | --- | --- | --- | --- | --- |
| T cell (CD8+) | CD45+ | TCRab+ | CD3e+ | CD8+ |  |
| T_RM_ | CD127+ | CD103+ |  |  |  |
| T_EM_ | CD127+ | Ly6C+ |  |  |  |
| T cell (CD4+) | CD45+ | TCRab+ | CD3e+ | CD4+ |  |
| Treg | CD25+ | FoxP3+ | CD38+ | ICOS+ |  |
| T_eff_ | CD127+ | Ly6C+ |  |  |  |
| B cell | CD45+ | CD11b- | CD19+ | B220+ |  |
| Naive B cell | CD43+ | IgD- |  |  |  |
| Mature B cell | CD43- | IgD+ |  |  |  |
| DC | CD45+ | CD11b+ | CD11c+ | MHCII+ |  |
| CD103+ DC | CD103+ |  |  |  |  |
| cDC | CD44+ | CD172a+ |  |  |  |
| MDSC | CD45+ | CD11b+ | MHCII- |  |  |
| Monocytic MDSC | Ly6C+ | F4/80- | MHCII- |  |  |
| CD11C+ MDSC | CD11c+ | MHCII- |  |  |  |
| Tissue-resident macrophage | F4/80+ | CD69+ | MHCII- |  |  |
| Monocytes | CD45+ | CD11b+ | Ly6C+ | F4/80+/- | MHCII+ |
| Macrophage | CD45+ | CD11b+ | Ly6C- | F4/80+ | CD69- |
| M1-like macropahge | BST2+ | MHCII+ |  |  |  |
| M2-like macrophage | Arg-1+ |  |  |  |  |

Table S3.

Summarization of markers used to identify each cell populations.

| No. | Days | Pathology | Metastasis (Y/N) |
| --- | --- | --- | --- |
| 1 | 39 | Normal | N |
| 2 | 37 | Normal | N |
| 3 | 37 | Normal | N |
| 4 | 33 | PanIN | N |
| 5 | 63 | Normal | N |
| 6 | 61 | ADM | N |
| 7 | 59 | PanIN | N |
| 8 | 74 | Normal | N |
| 9 | 108 | ADM | N |
| 10 | 106 | ADM | N |
| 11 | 89 | Normal | N |
| 12 | 120 | PanIN | N |
| 13 | 116 | PanIN | N |
| 14 | 60 | PDAC | N |
| 15 | 94 | PDAC | N |
| 16 | 167 | PDAC | N |
| 17 | 48 | PDAC | N |
| 18 | 61 | PDAC | N |
| 19 | 139 | PDAC | Y |
| 20 | 97 | PDAC | Y |
| 21 | 95 | PDAC | Y |
| 22 | 149 | PDAC | Y |

Table S4.

KPC mice overview.

| Group | No. | Age | Gender | Blood Type | Primary Disease |
| --- | --- | --- | --- | --- | --- |
| Donor Pancreas | **1** | 14 | Male | A, Rh+ | Cerebral hemorrhage |
|  | **2** | 40 | Male | A, Rh+ | Traumatic brain injury |
|  | **3** | 42 | Male | B, Rh+ | Traumatic brain injury |
|  | **4** | 44 | Male | B, Rh+ | Cerebral hemorrhage |
|  | **5** | 47 | Female | O, Rh+ | Cerebral hemorrhage |
|  | **6** | 44 | Male | B, Rh+ | Traumatic brain injury |
|  | **7** | 37 | Male | A, Rh+ | Traumatic brain injury |
|  | **8** | 42 | Male | B, Rh+ | Traumatic brain injury |

Table S5.

Clinical information of pancreas donors.

|  | No. | Age | Gender | Neoadjuvant therapy | Pathology | Differentiation | TNM Stage |
| --- | --- | --- | --- | --- | --- | --- | --- |
| RPC | **1** | 79 | Female | No | PDAC | Well | T1N0M0, Stage IA |
|  | **2** | 68 | Male | No | PDAC | Moderate-Poor | T1N0M0, Stage IA |
|  | **3** | 81 | Male | No | PDAC | Well | T3N0M0, Stage IIA |
|  | **4** | 68 | Male | No | PDAC | Moderate | T1N0M0, Stage IA |
|  | **5** | 56 | Male | No | PDAC | Well | T3N0M0, Stage IIA |
|  | **6** | 60 | Male | No | PDAC | Moderate-Poor | T2N0M0, Stage IB |
|  | **7** | 63 | Female | No | PDAC | Moderate | T3N0M0, Stage IIA |
|  | **8** | 67 | Female | No | PDAC | Moderate | T3N0M0, Stage IIA |
| MPC | **1** | 51 | Male | No | PDAC | Moderate | T3N1M1, Stage IV |
|  | **2** | 62 | Male | No | PDAC | Moderate | T3N1M0, Stage IIB |
|  | **3** | 61 | Male | No | PDAC | Moderate | T3N1M0, Stage IIB |
|  | **4** | 55 | Male | No | PDAC | Moderate | T2N1M1, Stage IV |
|  | **5** | 61 | Male | No | PDAC | Moderate | T2N1M0, Stage IIB |
|  | **6** | 64 | Male | No | PDAC | Moderate-Poor | T3N1M1, Stage IV |
|  | **7** | 48 | Female | No | PDAC | Moderate | T3N1M0, Stage IIB |
|  | **8** | 70 | Female | No | PDAC | Moderate | T3N1M1, Stage IV |

Table S6.

Clinical information of pancreatic cancer patients. TNM staging was based on AJCC 8^th^ edition.
